# Classification and Treatment of Diseases in the Age of Genome Medicine Based on Pathway Pathology

**DOI:** 10.3390/ijms22179418

**Published:** 2021-08-30

**Authors:** Iver Petersen

**Affiliations:** Institute of Pathology, SRH Poliklinik Gera, SRH-Wald-Klinikum Gera, Strasse des Friedens 122, D-07548 Gera, Germany; iver.petersen@gmail.com; Tel.: +49-365-828-6601; Fax: +49-365-828-6602

**Keywords:** Leonard Hayflick, Rudolf Virchow, Manfred Dietel, cellular pathology, predictive pathology, genome medicine, cancer biology, classification, treatment

## Abstract

The focus of pathology as a biomedical discipline is the identification of the pathomechanisms of diseases and the integration of this knowledge into routine diagnosis and classification. Standard tools are macroscopic and microscopic analysis complemented by immunohistochemistry and molecular pathology. So far, classification has been based on the paradigm of cellular pathology established by Rudolf Virchow and others more than 150 years ago, stating that diseases originate from diseased cells. This dogma is meanwhile challenged by the fact that cells can be fully reprogrammed. Many diseases are nowadays considered to originate from undifferentiated stem cells, induced into a diseased state by genetic or epigenetic alterations. In addition, the completion of the Human Genome Project, with the identification of more than 20.000 genes and a much higher number of gene variants and mutations, led to the concept that diseases are dominated by genetics/epigenetics rather than cells of origin. The axiom of cellular pathology, however, still holds true, as cells are the smallest animate units from which diseases originate. Medical doctors and researchers nowadays have to deal with a tremendous amount of data. The International Classification of Diseases will expand from 14.400 entities/codes in ICD-10 to more than 55.000 in ICD-11. In addition, large datasets generated by “genomics“, e.g., whole-genome sequencing, expression profiling or methylome analysis, are meanwhile not only applied in research but also introduced into clinical settings. It constitutes a major task to incorporate all the data into routine medical work. Pathway pathology may help solve this problem. It is based on the realization that diseases are characterized by three essential components: (i) cells of origin/cellular context and (ii) the alteration of cellular as well as (iii) molecular/signal transduction pathways. The concept is illustrated by elaborating on two key cellular pathways, i.e., the cellular senescence of normal cells and the immortality of cancer cells, and by contrasting single cell/single pathway diseases, such as mycoplasma and coughing pneumonia, with complex diseases such as cancer, with multiple cell types as well as multiple affected cellular and signaling pathways. Importantly, the concept of pathway pathology is not just intended to classify disease, but also to conceive new treatment modalities. This article is dedicated to Dr. Leonard Hayflick, who made basic discoveries in pathway pathology not only by identifying cells causing disease (Mycoplasma pneumoniae) and establishing cell strains for treating disease (WI-38 for viral vaccines), but also by first describing cellular senescence and immortality.

## 1. Introduction

### 1.1. Traditional and Emerging Ways of Classifying Diseases

Pathology is a basic medical discipline that has been particularly engaged in the visualization of diseased tissues and cells. In addition, it has an inherent mission to rationalize the underlying pathogenetic mechanisms of medical disorders. Its classical tools are macroscopy and microscopy (histology, immunohistochemistry and cytology) complemented by molecular analysis; in situ techniques in particular are used for the detection of genes and proteins in the context of diseased tissues. Molecular pathology using biochemical techniques for the analysis of DNA, RNA and protein extracts has profoundly extended the methodological repertoire. The use of these techniques has been highly successful in the classification of diseases, which is reflected in the WHO tumor classifications.

Histopathology, i.e., the microscopic evaluation of tissues, is still the gold standard for classification purposes, especially in tumor pathology. On the one hand, it can be used to access single parameters such as tumor grading. On the other hand, histopathology, in a similar manner to clinical inspection, represents a holistic approach. Morphology harbors complex information that can be analyzed comprehensively by the brain, often leading to a diagnosis within seconds just by the human’s exquisite capabilities in pattern recognition [1].

**Figure 1:**
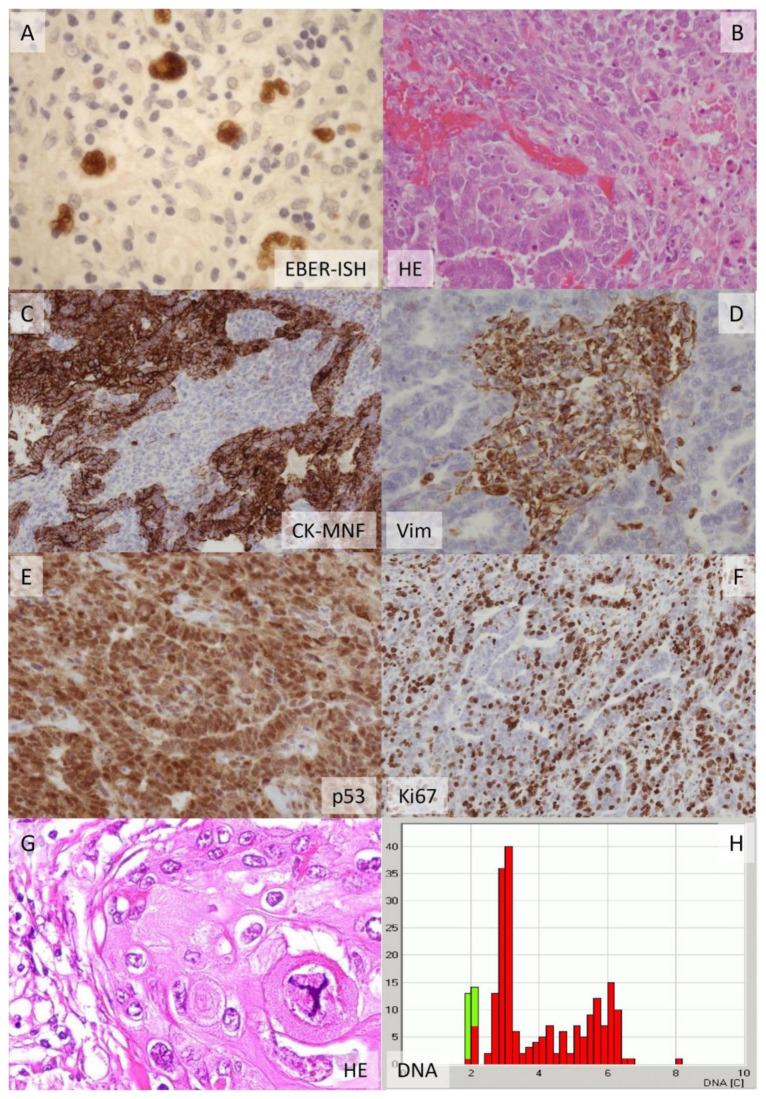
Examples of three tumor cases illustrating the power of histological and immunohistochemical analysis to identify the cellular origin of and active cellular and molecular pathways within malignant neoplasms. The large multicellular tumor cells of classical Hodgkin lymphoma (**A**) become visible via the positive EBV reaction (EBER-ISH) in a prominent background of leucocytes, highlighting that Hodgkin lymphoma is an “inflammatory” neoplasm being driven by NFkB pathway activation. The ovarian carcinosarcoma (**B**–**F**) is characterized by a glandular epithelial component and a sarcoma component (**B**), demonstrating a carcinoma that has undergone epithelial–mesenchymal transition (EMT). Furthermore, the tumor shows hemorrhage (left) and apoptotic bodies (right). The epithelial component becomes visible in in a cytokeratin (CK) reaction (**C**) while the mesenchymal component is revealed by a vimentin stain (**D**). The carcinosarcoma shows a positive p53 reaction (**E**) in the nuclei (and cytoplasm), which is characteristic for this TP53-mutated tumor entity. The proliferation (Ki67) is moderate to high (**F**). The non-small-cell carcinoma of the lung (**G**) harbors large non-small nuclei and large atypical tripolar mitosis, which is indicative of aneuploidy and specifically a DNA content (**H**) in the hyperdiploid range with a triploid stem cell line [6].

Hodgkin’s disease is an interesting example. It was long considered an inflammatory condition, potentially related to tuberculosis. This is understandable considering the microscopic picture, which is dominated by inflammatory cells such as lymphocytes and eosinophilic granulocytes together with giant and multinuclear cells. These were initially confused with epithelioid cells and giant cells typically and much more frequently found in tuberculosis or other granulomatous inflammatory conditions (Figure 1). Additionally, the clinical symptoms, including fever, may resemble an infectious disease. However, Carl Sternberg and Dorothy Reed later described in more detail the cells characterizing the disease, which were finally renamed Hodgkin cells and Reed–Sternberg cells. Thereby, it became clear that the autopsy cases which Thomas Hodgkin described in 1832 via macroscopy represented a neoplastic disease of the lymph nodes. His discovery laid the foundation for lymphoma classification which still differentiates between Hodgkin and non-Hodgkin lymphomas.

The disease is meanwhile curable by a combination of chemotherapy and radiation, and also constitutes a paradigm of a cancer entity which is highly sensitive to an intelligent combination of therapeutic regimens [2,3]. We meanwhile know that the disease, together with many other non-Hodgkin lymphomas, is molecularly dominated by a constitutive activation of the NFkB pathway [4,5].

### 1.2. Big Data in Disease Classification, Predictive Pathology and Precision Medicine

The paradigm of disease classification by humans is challenged by highly sophisticated molecular techniques employing whole-genome techniques, bioinformatical analytical tools and computer science. A first glimpse of the power of comprehensive tumor analysis already appeared in the era of comparative genomic hybridization (CGH) via the detection of recurrent chromosomal imbalances in solid tumor entities. CGH revealed repetitive patterns of DNA imbalances in many tumor types, which are associated with tumor types, grading and prognosis [7,8,9].

Whole-genome expression profiling then allowed the stratification of morphologically defined tumor types into prognostically relevant subtypes in breast or lung cancer, for example [10,11]. Recently, methylome profiling was used to identify more than 100 rare brain tumor subtypes which are difficult to classify by utilizing non-specialty pathologists [12]. The same methodology is also able to classify soft tissue tumors [13,14].

At the same time, classical pathology analysis using immunohistochemistry together with molecular pathology has entered another clinical arena, i.e., the selection of patients for specific “individualized” therapeutic approaches constituting the field of predictive pathology. In many instances, it is (still) sufficient to analyze a single or only a few parameters in order to decide on the existing treatment modalities [15]. This approach, being based on classical histopathology combined with immunohistochemistry and specific biomarkers, has been highly successful.

Others have propagated the term “precision medicine”, which is essentially based on a “shotgun” approach, i.e., the genome-wide investigation to find a mutation that might be targetable. Furthermore, this approach favors a histology-agnostic approach in the selection of therapeutics [16]. So far it has been far less successful than predictive pathology, and has only led to a very limited number of FDA and EMA approvals for cancer therapies.

Despite the power of modern techniques in analyzing diseases, one should be aware of their limitations and the challenges that need to be tackled. Medical doctors and scientists are confronted not only with an impressive number of diseases. The International Classification of Diseases harbors 14400 codes in ICD-10 and this number will expand to 55.000 entries with ICD-11, which will become operative in 2022 (https.//www.who.int/health-topics/international-classification-of-diseases (accessed on 13 June 2019)).

In addition, they are confronted with more than 20.000 genes [17]. Considering genetic variants, e.g., mutations, polymorphisms and splicing variants, in addition to non-coding RNAs, such as microRNAs, involved in gene regulation, the numbers can easily be multiplied. These numbers are daunting and most humans will be appalled and may not even consider approaching the endeavor of knowing and understanding all genes and their relevance for diseases. However, this may underestimate the capacities of the human brain. In fact, Shakespeare used more than 31.500 different words in his collected writings and was considered to know about 66.500 words [18]. Thus, the human brain has impressive capabilities for storing and handling information.

A defined terminological system is highly relevant for standardized data collection. The term “ontology” serves this purpose. The term originates from metaphysics/philosophy, dealing with the (fundamental) nature of being. In modern times, it was adopted to define a set of concepts and categories in a subject area that show properties and to define relations between them. Ontology is thereby used for classification purposes. For instance, “Gene Ontology (GO)” has been introduced by bioinformaticians to describe gene functions [19] (see Section 2.3 “Cellular Pathways”).

The ontology concept was also adopted for clinical purposes, e.g., by the Consortium of Brachytherapy Analysis (COBRA) to define and categorize terms for the standardized evaluation of the effect of brachytherapy in head and neck and skin cancer; the initiatives were termed ENT COBRA and SKIN COBRA [20,21]. A set of 240 and 290 terms were defined in the ENT and SKIN ontology projects, respectively. This seems more manageable than the high number of genes and ICD codes. It still represents an enormous task in clinical practice as all these parameters need to be documented and digitized in a standardized way. The COBRA ontologies so far do not seem to incorporate molecular data of cancer specimens.

### 1.3. International Classification of Diseases: ICD, ICD-O and TNM

The International Classification of Diseases (ICD) provides a scaffold for categorizing maladies. It employs subcategories that group disorders according to the organ system being affected, e.g., the heart and vascular system, infectious diseases or metabolic disorders. These all represent separate chapters of the ICD system. Thus, it is related to clinical subspecialties in medicine, such as cardiology, infectiology, endocrinology, hematology, etc. There are, however, diseases related to several organs, such as neoplasms or immune disorders (and others), that constitute additional distinct chapters in the ICD. ICD-11 will become “all-inclusive”, as symptoms as well as clinical and laboratory findings will become a part of this classification (Table 1).

Furthermore, ICD-11 was conceived in such a way that any disease, its severity and also its treatment could be incorporated into the ICD system. For several diseases, such as neoplasia, this may be accomplished by incorporating already-established classification systems, the ICD-O (O = oncology) and TNM systems, into ICD-11. Both are the basis for the diagnosis and treatment of neoplastic diseases. The advantage of ICD-O is that it provides information on the site and cell of origin of the disease, while TNM offers information on some of its biological characteristics including tumor extent, lymph node metastasis and hematogenous dissemination.

In ICD-11, a chapter on traditional medicine was introduced. In addition, not only may diseases be coded, but the severity of disease states, histological entities and medications may also be. This can be done on the one hand by the new chapters V (supplementary section for functioning assessment, see No. 27 in Table 1) and X (No. 28, Table 1), which harbors “extension codes”. On the other hand, a content model with a list of fields for each entity has been introduced in ICD-11 (Table 2). However, the content model is far from complete for most ICD-11 entities, and in all likelihood will never be completed [22].

More than 5400 rare diseases coded in the French–European Orphanet were included in the ICD-11 classification. They were distributed in most “organ chapters” and not just chapter 20, which dealt with developmental anomalies [22].

The ICD system had gained considerable momentum with the introduction of disease-related groups (DRGs), which have become the basis for recompensation purposes of hospitals and medical doctors in many countries. In doing so, it has generated an impressive wealth of data. However, it probably will not (and is not intended to) advance biomedical science and the understanding of disease mechanisms. In the author’s view, it is problematic that the ICD system may consolidate a traditional view on medicine. The ICD codes are also used to implement quality assurance programs in medicine, again favoring convention over innovation.

## 2. Pathway Pathology—The Key Principles

Pathway pathology is intended to provide an intellectual scaffold for integrating genetic, molecular and biological information into disease classification and treatment, enabling medical doctors and scientists to grasp and incorporate the wealth of data on diseases and diseased people for the benefit of individual patients.

**Figure 2:**
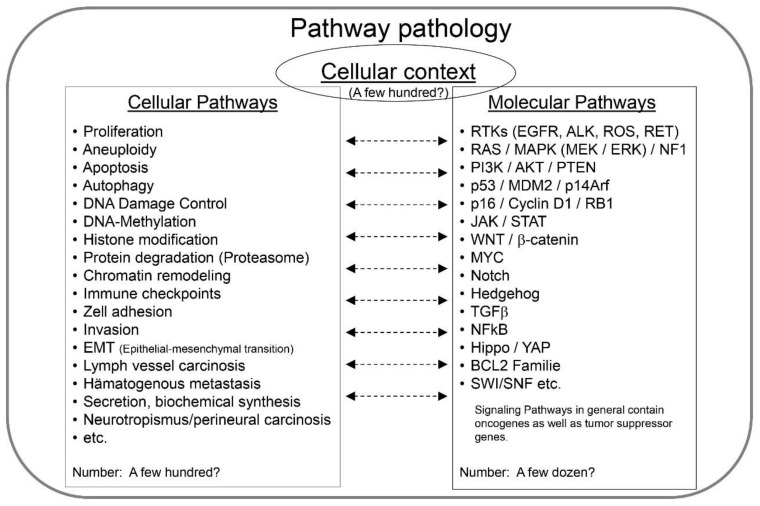
The pathway pathology of any disease can be rationalized by the primary cells and their cellular context that initiates the disease as well as the molecular pathways (genes and signaling pathways) and cellular mechanisms/pathways driving it. Knowing these components and their relevance is essential not only for a correct classification but also for an effective therapy. This is particularly true for complex diseases, such as cancer, that often carry multiple mutations and many active cellular mechanisms.

The basic concept of pathway pathology is the idea that diseases can be characterized by integrating knowledge of three fundamental components, i.e.,

(1) The cellular context of the disease;
(2) The genes and proteins being affected, i.e., the molecular pathways;
(3) The cellular mechanisms being altered, i.e., the cellular pathways (Figure 2).

### 2.1. Cellular Context

This axiom dates back to the times of Rudolf Virchow and the introduction of the concept of cellular pathology in 1858 [23]. Virchow propagated and popularized the fact that diseases originate from cells that are malfunctioning. Each biological process is operating in a cellular context. The cell is the smallest unit within a living organism with the capabilities of self-renewal (“omnis cellula a cellula”) and propagation of biological information. This axiom still holds true despite the extensive knowledge on molecular alterations and the deciphering of the human genome.

Recently, Virchow’s “Zellularpathologie” has received regained interest and momentum by the characterization of stem cells and their roles in embryonic development and tissue renewal as well as by the introduction of the concept of cancer stem cells being considered key players in the progression of malignant diseases [24]. The generation of pluripotent stem cells from adult somatic cells by the introduction of specific genes, i.e., the Yamanaka factors OCT4, SOX2, MYC and KLF4, was a major step in the understanding of stem cell biology. It revealed that potentially any cell can be reprogrammed by appropriate genetic/molecular alterations [25]. This discovery somehow weakened the “old” concept of cellular pathology since the potential of cellular reprogramming of any somatic cell questioned the importance of the cellular origin of a specific disease. However, daily clinical practice and numerous scientific publications have strengthened the significance of specific cells as origins of disease. This may be best illustrated by the example of infectious diseases which strictly require the presence of non-human cells or agents.

**Figure 3:**
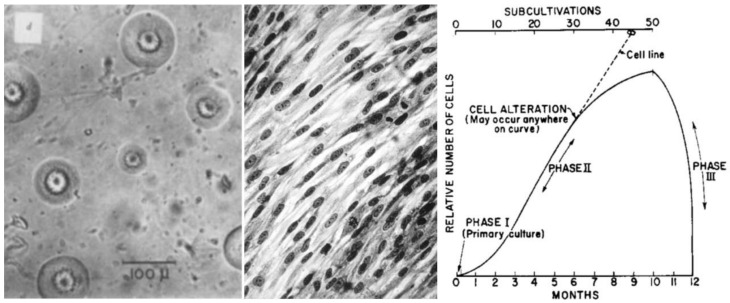
Mycoplasma pneumoniae is one example of a non-human cell that is capable of causing disease, i.e., coughing pneumonia, as shown by Leonard Hayflick more than 60 years ago. In those days the organism was called pleuropneumonia-like organism (PPLO); it shows a characteristic colony growth in culture (left, image taken from Hayflick and Stinebring 1960 [27]). Hayflick also established the first normal cell strain (WI-38, middle, image courtesy of Dr. Leonard Hayflick), which he distributed worldwide, thereby becoming the source of most viral vaccines. At about the same time, Leonard Hayflick and Paul Moorhead used similar cell cultures to characterize replicative cellular senescence for the first time; the “Hayflick limit” was born (right, image taken from Hayflick and Moorhead 1961 [29]).

Coughing pneumonia is one example. This atypical pneumonia was known for a long time and it was speculated that the origin of the disease was a virus. Leonard Hayflick was the first to discover that the organism (Figure 3) represents an unusually small bacterium that grows intracellularly in tissue culture, contaminates these cultures and causes the disease [26,27,28].

Cellular context is also highly relevant for cancer as it has been convincingly shown that the effect of tumor-causing mutations is highly dependent on the type of cell in which the genetic defect is introduced [30,31]. In addition, the behavior and in particular the response to cancer treatment is highly dependent on the tumor microenvironment being composed on a multitude of non-cancer cells [32]. Similarly, only certain cell types are susceptible to specific microorganisms and viruses representing targets for infection. Finally, the knowledge of cellular context and the choice of right cells are also highly relevant for the development of efficient therapies.

Again, Leonard Hayflick made seminal contributions in this regard by generating the WI-38 cell strain (Figure 3), the first non-cancerous and non-virally contaminated human cells that could be used for the generation of vaccines. Apart from producing the first vaccine in this cell culture model [33], he ensured that these cells became accessible to investigators worldwide, including pharmaceutical companies that used them for vaccine production. Thereby, the cells became the standard vehicle for vaccine production against multiple viruses [34].

Cellular context is of eminent importance in disease classification and therapy. Interestingly, even more than 160 years after Virchow established the axiom of the cellular origin of disease, in many disorders the progenitor cell has not yet been unequivocally identified. In addition, the identification and typing of specific cell types in humans and other species seems far from complete.

There is a Wikipedia website listing distinct cell types of the human body, i.e., https://en.wikipedia.org/wiki/List_of_distinct_cell_types_in_the_adult_human_body (accessed on 4 July 2021) (Google: “cell type human body”). It contains around 230 different cell types. However, this list is not fully comprehensive and is not yet related to disease entities. Dendritic cells, for instance, are mentioned as cells of the lymphoid tissue. However, the Langerhans (dendritic) cell which is found in the skin, in addition to other organs, and linked to Langerhans cell histiocytosis (LCH) is not mentioned. Interestingly, LCH is a neoplasm that occurs in many tissues and is associated with a characteristic genetic defect, i.e., MAPK pathway activation by BRAF V600E or other mutations. The disease is amenable to targeted therapy with appropriate inhibitors [35].

### 2.2. Molecular Pathways

The transmission of biological information within a cell and between cells is highly organized and dependent on ligands, receptors and signaling pathways. Many such signaling pathways have meanwhile been identified and well characterized. In addition, signaling pathways are major constituents of databases such as the Kyoto Encyclopedia of Genes and Genomes, KEGG (see www.wisdom.weizmann.ac.il (accessed on 4 July 2021) or Google: “signal transduction pathways databases”). Biomedical companies including Cell Signaling Technology (www.cellsignal.com (accessed on 28 August 2021)) are focusing on these pathways and providing reagents for their investigation.

Importantly, these molecular pathways constitute ordered frameworks of interactions of proteins and thereby provide a structure for a considerable number of genes. However, many genes and their variants are involved in different signaling pathways. The p53 protein constitutes an important example. It is listed in 61 different map entries of the KEGG database, ranging from the p53 signaling pathway itself over the MAPK, PI3K-Akt, WNT and HIPPO signaling pathways to several different cancer type pathways as well as apoptosis, cell cycle, cellular senescence, ferroptosis, autophagy and metabolic pathways to non-cancer diseases including amyotrophic lateral sclerosis, Huntington’s disease, measles and HSV and EBV infection (see KEGG pathways).

Adding another level of complexity is the fact that the signaling pathways are linked with each other and constitute flexible networks. Thus, the many static schemata of specific pathways constitute only rough estimations of the molecular signaling at work in specific disease states. Furthermore, these schemes usually apply only in certain circumstances and defined cell types. Still, it is important to realize that they constitute basic molecular networks that are relevant for all cells. In addition, as exemplified for p53, they are relevant for many different cellular mechanisms and diseases.

Many genes do not constitute a component of specific signaling pathways. However, due to potential protein–protein interactions (see, e.g., https://www.ncbi.nlm.nih.gov/gene (accessed on 4 July 2021)), it is likely that further genes will be linked to already characterized signaling pathways. Furthermore, it is important to realize that many more genes are regulated by defined signaling pathways as there are specific promoter sequences, including the TP53 binding site, etc., within the genome. Thus, the expression of specific genes might be informative for identifying whether a specific signaling pathway is active or not. This is just a reminder of the fact that cellular signaling occurs in a hierarchical fashion, e.g., extracellular stimuli are transmitted to membranous or intracellular receptors that transmit the signal to the cell nucleus by the activation of transcription factors such as TP53, MYC, SMAD4 or many others.

Although there might be signaling pathways that have not been identified yet, many are meanwhile well-characterized and it is important for biomedical researchers and medical doctors alike to become familiar with these regulatory pathways, of which some are listed in Figure 2.

### 2.3. Cellular Pathways

Although many genes have not yet been functionally analyzed, each is supposedly linked with a purpose and at least one cellular function. These cellular mechanisms constitute another framework for understanding and grouping genes. There is a considerable number of cellular mechanisms which however should be smaller than the number of genes. Figure 2 lists only few of them; some cellular pathways probably still await discovery.

Several databases as well as intellectual and ontological approaches have been developed for their categorization, e.g., BioSystems pathways—KEGG, WiKiPathways, REACTOME—and Gene Ontology (see https://www.ncbi.nlm.nih.gov/gene (accessed on 4 July 2021)). The Gene Ontology Consortium was established in 2000 and represents the most comprehensive approach for describing genes and gene functions [36]. There has been an impressive increase in the number of GO terms between 2002 and 2010, amounting to almost 7000 entries for homo sapiens and even higher number for other organisms. This number seems far too large to be easily manageable by humans. Indeed, the primary purpose of the GO initiative was to make gene information manageable for computational analysis by bioinformaticians [19].

In comparison, the KEGG database is far less comprehensive. It provides maps for roughly 50 cellular processes and in addition 12 metabolic pathways and 30 signal transduction pathways (see www.kegg.jp (accessed on 4 July 2021)). Interestingly, KEGG also provides databases on chemical information (KEGG COMPOUND, KEGG GLYCAN, KEGG REACTION and KEGG ENZYME) as well as health information (KEGG NETWORK/VARIANT, KEGG DISEASE, KEGG DRUG and KEGG ENVIRON). The data is manually curated and KEGG has been initiated already in the year 1996 [37].

**Figure 4:**
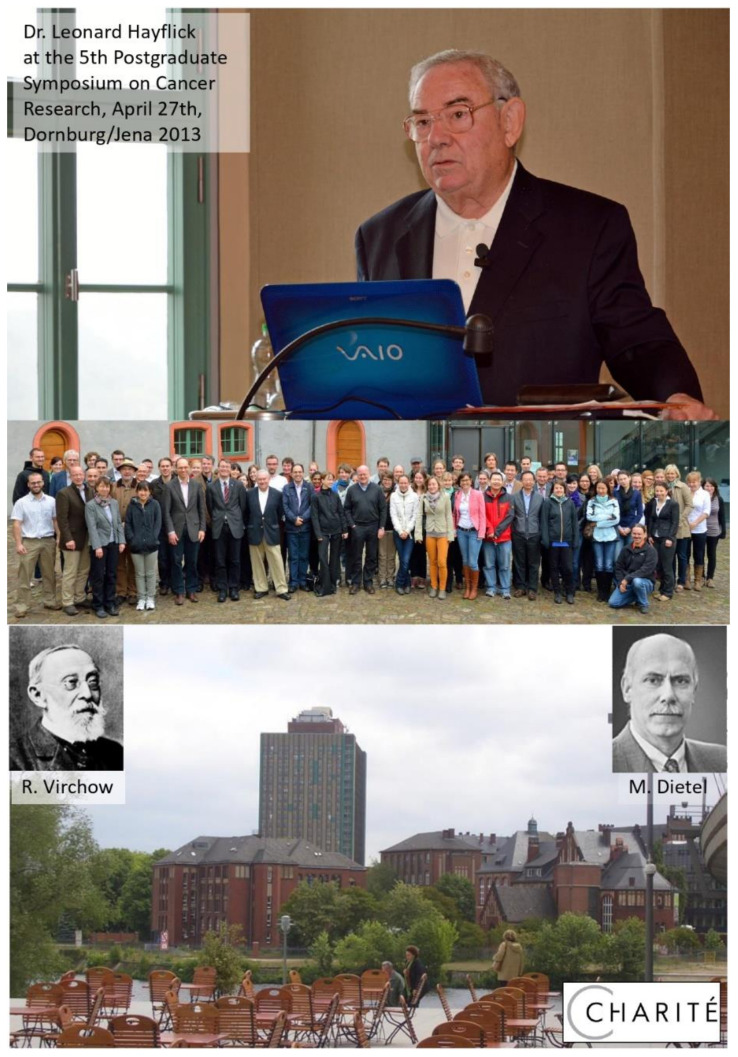
This article is dedicated to Dr. Leonard Hayflick, shown here during his lecture at the 5th postgraduate cancer research symposium in the old castle of Dornburg, which is located close to Jena in Thuringia/Central Germany. Rudolf Virchow established the concept of cellular pathology in 1858 during his Würzburg period. Due to his reputation as both a scientist and a politician he was able to propagate his concept worldwide and build a large pathology institute at the Charité University Hospital in Berlin. This article is also dedicated to Dr. Manfred Dietel, who was the successor of Rudolf Virchow between 1994 and 2016, after the German reunification. The picture shows the “Rudolf-Virchow-House” and the Charité “skyscraper” in 2006, as seen from the terrace of the new Berlin Central Station.

The listing of cellular pathways in Figure 2 only represents a limited number of mechanisms that need to be considered. Again, Leonard Hayflick made seminal discoveries by identifying essential pathways in cell biology and medicine, i.e., cellular senescence of normal cells after a limited number of cell replications/passages (the Hayflick limit, see Figure 3) and immortality of cancer cells [29,38]. The Nobel prize in physiology and medicine in the year 2009 was given to Elizabeth Blackburn, Carol W. Greider and Jack Szostak for the discovery how chromosomes are protected by telomeres and the enzyme telomerase. Their important discoveries revealed essential components of the cellular pathway of replicative cellular senescence and were enabled by the cell model, tools and knowledge generated by Leonard Hayflick (Figure 4) almost half a century before. Unfortunately, his contributions were not recognized.

## 3. Pathway Pathology in Research and Clinical Practice

### 3.1. Immortality and Cellular Senescence Represent Key Cellular Pathways

Immortality and cellular senescence are particularly important in cancer and stem cell biology. In addition, both represent paradigms as to how cellular pathways are related to distinct molecular mechanisms, leading to a subtyping of these cellular pathways. For instance, immortality can be achieved by telomerase activation, but also by the ALT (alterative lengthening of telomeres) mechanism [39]. Cellular senescence is even more complex, being meanwhile subdivided into replicative senescence, oncogene-induced senescence (OIS) and developmental senescence [40,41,42].

In this context, it should be mentioned once more that the outcome of genetic alterations on cellular pathways is dependent on the cell type. OIS may serve as an example. It was first described by transfecting an activated HRAS vector (H-rasV12) into rodent and human fibroblasts, Hayflick’s WI-38 cells among others [43]. In contrast, OIS induction in melanocytes was done by BRAF V600E, in epithelial cells by KRAS G12V and by NRAS G12D in lymphoid cells [44]. This again indicates that cellular background matters greatly and needs to considered when analyzing the effects of genetic alterations. The common denominator for successful OIS induction seems to be the (over)activation of the Raf/Mek pathway, however, other molecular pathways are also involved like PI3K/AKT/mTOR, VHL/HIF and β-catenin [44].

### 3.2. The Interplay between Molecular and Cellular Pathways in Specific Cells Predicts Biological Behavior

Considering cancer as a multigenetic disease, there are several essential cellular pathways that have already been identified, i.e., the so-called “hallmarks of cancers” [45]. In essence, all of them constitute cellular mechanisms that are affected in different tumor subtypes to different extents. Metastasis is fortunately not active in all cancer types, but if active it heavily dominates the prognosis. Similarly, cellular proliferation/cell cycle activation differs largely between highly aggressive tumor types such as small cell lung cancer (SCLC) or diffuse large B-cell lymphoma (DLBCL) on the one hand, and well differentiated ones such as squamous cell carcinoma (SqCC) of the skin (spinalioma) or indolent follicular B-cell lymphoma on the other. The cells of origin of these neoplasms are well established, i.e., epithelial cells of the lung airways or the skin and B cells of lymphoid tissues. In contrast, for other cancer types like synovial sarcoma or Merkel cell carcinoma, the cells of origin are still debated and their clinical behavior is much more difficult to predict [46,47].

To understand these neoplasms and to predict their clinical behavior, it is essential to know which signaling pathways, respectively, are responsible for which cellular pathways and their relative strength of activation in the relevant cell type. For instance, strong MYC activation by gene amplification or other genetic mechanisms seems to be essential for high-proliferation cancer types such as SCLC or DLBCL (with a dependency on apoptotic cell signaling), whereas MAPK kinase activation is associated with comparatively lower cell cycle activation [48,49,50].

The molecular basis of many cancer hallmarks has already been resolved or will be known in the future. But many questions remain. An imminent one is related to the analytical tools and diagnostic measures that should be applied to pinpoint the key components of diseases.

### 3.3. Analytical Tools in Pathway Pathology—Whole-Genome Analysis

The proponents of systems biology/pathology and Gene Ontology, as well as major computational companies such as Apple, Google and alike, argue that the acquisition and analysis of large-scale datasets by bioinformatical tools and apps are important for the understanding and treatment of disease. Indeed, there is no doubt that the interrogation of diseased human beings and tissues have enhanced our knowledge on the molecular pathology of many maladies.

Gene expression profiling by cDNA microarrays and Affymetrix chips represented the first genomic tools to analyze almost the entire genome by gene resolution. With respect to breast and lung cancer, it confirmed and refined the histological tumor classification [10,11]. Specifically, it identified subsets of lung adenocarcinomas carrying distinct prognoses [10]. This was later recapitulated morphologically and led to a refined histological grading system that was included into the IASLC and later the WHO classification of lung cancer [51]. Furthermore, in breast cancer, the subtyping of luminal A, luminal B, basal subtypes, etc., can be done by classical techniques. However, gene panel analysis has meanwhile been firmly introduced in breast cancer diagnostics to better stratify patients who benefit from adjuvant chemotherapy from those that don’t [52].

Another genome-wide analysis has meanwhile entered clinicopathological practice. Whole-genome methylation analysis combined with bioinformatical analysis has recently been shown to be able to classify more than 100 different brain tumor subtypes [12]. In a similar manner, this technique is able to correctly classify up to 100 rare soft tissue tumor entities [13,14]. In addition, this technique is helpful in identifying carcinomas of unknown primary sites [53]. It may also be able to predict therapy responses of lung cancer patients treated with immune checkpoint inhibitors [54,55].

Since soft tissue and neuropathological tumor pathology is a field for expert pathologists, these developments can be viewed as breakthrough discoveries in cancer classification as they show for the first time that whole-genome analysis may be better than normal pathologists in determining cancer subtypes. This technique will now be applied to the subtyping of carcinomas which represent much more frequent tumor entities than sarcomas and brain tumors. In addition, the technique provides a gene copy number profile that by itself has potential predictive power for therapy (e.g., HER2 amplifications) and prognosis [14].

Whole-genome mutation analysis, in contrast, has only minimal power in the classification of cancer subtypes because most gene mutations are not specific for a single entity; KRAS or p53 constitute good examples. There are a few exceptions, e.g., the identification of activating mutations in exon 19 or exon 21 of the EGFR gene is not only highly relevant for treatment selection but may become diagnostic if detected by a “liquid biopsy” from the blood of a patient with a radiologically detected bronchopulmonary neoplasm [56].

Next-generation sequencing (NGS) merits some consideration as it represents the basic tool for deciphering the human and other genomes and is meanwhile widely used for diagnostic and predictive purposes [56]. Similar to methylome analysis, NGS or deep sequencing may provide a gene copy profile of a sample. However, its major purpose is the sensitive detection of nucleotide exchanges as well as the interrogation of large stretches of DNA or RNA, providing data on tumor mutations as well as tumor mutation burden (TMB). Meanwhile, it has become a routine ancillary analysis in many clinical cancer trials and TMB may become a biomarker for immune checkpoint therapy as the extent of tumor mutations seems to have an impact on the number of neoantigens and thus the antigenicity of a tumor which can be attacked by the immune system [57].

TMB also highlights an underrecognized fact in cancer biology, i.e., that most malignant neoplasms are not characterized by a single mutation but instead dozens or even hundreds or thousands of mutations [58]. The challenge for molecular biologists, pathologists, oncologists, etc., nowadays is not to analyze and identify the mutations of a tumor sample but rather to pinpoint their relevance for cancer biology. What are the driver and what are the passenger mutations? Which ones should be targeted and which ones may be irrelevant?

Exactly at this point, pathway pathology may help because the mutations need to be interpreted within the cellular context of the disease and the cellular mechanisms that are active.

There are meanwhile several databases that may help with the interpretation of the biological significance of a specific mutation, e.g., the ones of the Sanger center or the Broad Institute [59].

### 3.4. Single Genes in Pathway Pathology

#### 3.4.1. The Case of TP53

TP53 is the single most frequently mutated gene across all cancer types, clearly highlighting its importance in cancer biology. The IARC, under the directorship of Paul Kleihues, established a mutation database more than 20 years ago [60]. TP53 mutations were associated with specific types of carcinogen exposure [60,61]. In 1993, it became the science molecule of the year [62]. Its discovery dates back to the year 1979 when a 53 kDa protein was identified as an essential factor for cellular transformation by the SV40 and polyoma virus on the one hand, and as a target of a humoral response against SV40-infected mice and serum antibodies in human breast cancer patients on the other hand.

The gene changed its status from an oncogene to a tumor suppressor gene and back to a bimodal tumor-supportive or tumor-suppressive gene dependent on its mutational status. Being involved in many cellular pathways as diverse as stress response, fertility and aging [63], it is the gene with the highest number of publications [64]. Still, it is not possible to reliably predict the biological activity of all p53 mutants and variants. In his 30th anniversary account on the history of p53, Thierry Soussi nicely illustrates how the research and insight regarding this gene was heavily influenced by dogmas and paradigm shifts [63]. He had the right foresight that the elucidation of p53 functions is not finished and that future birthdays will provide additional insight into the gene, which for instance happened with the discovery of its role in ferroptosis as a tumor-suppressive mechanism [65].

Despite the long history of TP53, mutation analysis of the gene has not become a routine diagnostic procedure in tumor classification as there is no cancer therapy yet approved that is dependent on the TP53 mutation status. However, it is probably just a matter of time until this will happen as there are meanwhile not only several oncogenes that are routinely assessed in this regard, e.g., EGFR and ALK in non-small-cell lung cancer or HER2 in breast cancer, but also tumor suppressor genes such as BRCA1 and BRCA2 that are also relevant for the selection of PARP inhibitors in ovarian cancer. In addition, there are many efforts under way for a TP53-based cancer therapy and it is important to note that TP53 mutations may tell us a lot about the biology of a specific tumor because there are already so many publications and data available on this gene [66,67,68,69].

TP53 remains highly fascinating as there are so many biological and molecular pathways associated with it. In cancer, one simple distinction relates to the fact that mutations may not only inactivate the tumor-suppressive function of the protein, the so-called “loss-of-function“ (LOF) alterations, but they may also induce new protumorigenic capabilites by which the gene may become as oncogenic as a bona fide proto-oncogene by “gain-of-function“ (GOF) mutations [68,70].

Mutation analysis alone is probably not able to fully predict the functional consequences of a certain gene alteration. As already outlined for other genes, the impact of a given p53 mutation may depend on the cellular context. Interestingly, there are certain correlations between the extent, strength and localization of the protein expression and p53 function. Ubiquitous and strong nuclear expression is the most frequently observed expression pattern that can be associated with an oncogenic GOF mutation (Figure 1). In contrast, inactivating single nucleotide mutations in one allele may be associated with a deletion of the second p53 allele, leading to a complete LOF mutation with concomitant loss of tumor-suppressive activity and complete loss of expression of the gene. Subtle to moderate and non-ubiquitous protein expression, e.g., at the invasion front, usually indicates a functional p53 gene being activated by cellular stress, such as proliferation. Furthermore, the cellular localization of the protein may suggest specific functional activities. For instance, cytoplasmic p53 expression may indicate an inhibitory effect on autophagy or trigger one on apoptosis [71].

Mutated TP53 is a “Pandora’s box”. Initially it seemed quite unlikely that such a small protein could have so many different functions and such widespread mutations, being detectable in nearly all tumor types. However, scientific evidence meanwhile overwhelming displays that all these effects are real. It is probably exactly this “functional omnipresence” together with its “non-essentiality” that makes p53 such an important player in (cancer) biology.

Most other cancer-associated genes are not able to interfere with as many cellular and molecular pathways as p53 is. But it is exactly the plethora of p53-associated cellular pathways that places the gene in the center of tumor biology, as so many cellular mechanisms need to be altered to create a highly malignant neoplasm. This requires reprogramming of cell growth, proliferation, migration, metabolism, angiogenesis, etc. This is essentially achieved by inducing changes in gene expression, which is a major function of p53.

Similarly potent alterations in cancer-associated genes, e.g., MYC amplification (neuroblastoma), SMARCB1 deletion (malignant rhaboid tumor), BRD4-NUT fusion and EWSR1 fusions also profoundly affect gene expression. In contrast, mutations in benign or low-grade tumors such as FGFR3 (seborrhoic keratosis and urothelial papillary tumors), BRAF (melanocytic nevi, Langerhans cell histiocytosis) and protein kinase C (benign fibrous histiocytoma) affect only single pathways and thus have far more restricted consequences for cellular homeostasis [72]. Along similar lines, (p53 wild-type) lung cancer with EGFR, ALK or ROS1 mutations has an inherently better prognosis than lung cancer with TP53 mutations, as the latter are frequently associated with additional genetic alterations, chromosomal instability and aneuploidy, generating the genetic plasticity needed for a highly malignant phenotype [73,74].

Targeting a single gene has been highly successful in cancer types that are virtually dependent on single mutations, such as GIST with c-kit mutations or CML with BCR-ABL fusion. Metastasis and drug resistance usually develop when additional alterations occur and chromosomal instability starts [75].

Unfortunately, there is still a large discrepancy in the number of publications on single genes and certain highly investigated ones, such as TP53 [64].

#### 3.4.2. S100A14

It is clear that gene products, apart from cells, constitute the major ingredients of pathway pathology as they are the basic components of molecular and cellular pathways. Meanwhile, all genes of the human genome are sequenced, but not all of them have been genetically and functionally analyzed which, however, is necessary to reveal their relevance in pathway pathology. Looking at two relatively unpopular genes is quite informative in this regard.

Our group, in collaboration with Claus Heizman and Beat Schäfer, first cloned and characterized the S100A14 gene [76]. It is a member of the S100 gene family, which constitute calcium-binding proteins, located in a gene cluster on chromosome 1q21. In total, 56 references are linked to this gene in the NCBI database GENE (as of July 4th 2021), mostly describing a connection with cancer. We identified the gene in this context using suppression subtraction hybridization for the comparison of lung cancer cells with normal bronchial epithelial cells. The overall picture of S100 proteins is, however, much broader, as they belong to three gene families, together with filaggrins, which are all located on the same chromosome regions on 1q and are linked with epithelial barrier functions that are essential for normal skin function and are altered in common diseases like atopic dermatitis and psoriasis/ichthyosis [77]. Looking at the gene and its genomic and functional associations thus opens up new perspectives for distinct pathways, diseases and tissue/cell types that might be affected by a malfunctioning of the gene.

#### 3.4.3. GABARAP

In a similar way, we described the GABARAP gene as a tumor suppressor gene on chromosome 17p, which is deleted in a p53-proficient breast cancer cell line [78]. Transfection and overexpression of the gene reduced tumorigenesis of the cell line in nude mice and was associated with the appearance of cytoplasmic vacuoles, suggesting a functional role in this cellular compartment. The gene was originally identified as a trafficking molecule for GABA A receptors in neurons. Later on, it was shown to be a homolog of the autophagy-related gene 8 (ATG8), belonging to the ubiquitin-like gene family, with LC3 being the most well-known representative.

We later studied the role of GABARAP in a knockout mouse model which surprisingly revealed that deletion in normal cells attenuated tumor growth both in a chemical carcinogenicity assay using DMBA as a (breast epithelial) carcinogen in the mouse model as well as after engrafting syngeneic melanoma cells in the knockout mice.

Two different mechanisms seem to be operative. In the carcinogenicity assay, the genotoxic stress from DMBA induced the expression of Xaf-1 in breast epithelial cells that probably triggered cell death, thereby inhibiting the propagation of mutated cells into cancer cells. In contrast, in the syngeneic tumor cell engraftment system, elevated cytokine expression of IL-1β, IL-6, IL-2 and IFN-γ in GABARAP KO immune cells probably inhibited tumor cell growth [79]. Thus, GABARAP seems to have different functions in epithelial and non-epithelial cells as well as normal and cancer cells. Again, cellular context matters. These seemingly contradictory observations, GABARAP promoting tumorigenicity in normal (murine) cells, but inhibiting tumor growth in (human) breast cancer, correlates with the fact that autophagy as an essential cellular pathway can act oncogenic as well as tumor suppressive [80].

Again, it is important to evaluate the full picture and to consider all three facets of pathway pathology. These two examples may illustrate how looking at single genes can be instructive in characterizing cellular and molecular pathways that might be ultimately exploited for the development of new therapies.

### 3.5. Impact of Cellular Pathology and Histopathology on Pathway Pathology

The concept of cellular pathology as proposed by Rudolf Virchow highlighted the importance of single cells as basic units of life and disease. In practical terms, however, it was essentially practiced in the form of histopathology, i.e., the analysis of tissue sections derived from disease specimens and its comparison with normal tissue. Histopathology became the basis for classification and still remains the gold standard for many diseases. This, however, is meanwhile challenged in many cases by clinical and basic science.

For instance, the detection of EGFR mutations in cell-free DNA in the blood (the so-called “liquid biopsy”) of a patient with a lung tumor is meanwhile almost equivalent to the diagnosis of a lung adenocarcinoma, as specific activating EGFR mutations are almost exclusively found in these tumor types [56]. A tissue biopsy of the tumor is no longer required to make this diagnosis.

Unfortunately, the power of classical histopathology and cytopathology is frequently overlooked and underestimated in the era of modern, new technologies. Microscopy can reveal the cellular origin of disease as well as the cellular pathways being affected. Figure 1 provides an example of this. It shows an undifferentiated tumor with features of a carcinosarcoma. As we meanwhile know by genetical analysis of tumor mutations, the malignant mesenchymal component of the tumor, the sarcoma, is derived from the epithelial component, the carcinoma, by the process of the epithelial–mesenchymal transition. Many different tumor entities are characterized by this phenomenon, e.g., metaplastic carcinoma of the breast, pleomorphic carcinoma of the lung, sarcomatoid carcinoma of the kidney and others. The biological similarity of these entities is not yet fully recognized, but it is likely that they are driven by common cellular and molecular pathways.

As another example, many tumor entities are defined by a striking inflammatory infiltrate, e.g., Hodgkin lymphoma in lymph nodes, giant cell lung carcinoma, medullary breast carcinoma and inflammatory myofibroblastic tumor of soft tissue. The inflammation is mediated by the activation of specific molecular pathways that cumulate in the secretion of cytokines and chemokines that boost inflammation and leukocytic infiltration. The clinical presentation of the disease, e.g., fever, leukocytosis and weight loss (the so-called “B-symptoms”) or cachexia, is also mediated by these molecular pathways. Thus, pathway pathology can be applied to explain and understand single parameters of a disease, and is not dependent on whole-genome analysis.

Similarly, the analysis of single biomarkers such as p53, EGFR or SOX2 in cancer samples can reveal a substantial amount about the pathway pathology of the disease [81,82].

Other examples for the power of histology and cytology to reveal cellular pathways is the detection of perineural tumor spread and vascular invasion, both constituting cellular mechanisms that have been extensively studied on the molecular level and are dependent on distinct molecular pathways [83,84].

Cytological features including size and type of mitosis or nuclear size and its variability (Figure 1) can be viewed as morphological biomarkers for the ploidy of a tumor cell and the presence of chromosomal instability [6,85]. There is, meanwhile, extensive data on the molecular processes that are linked to these cellular pathways and even the potential of using them as therapeutic targets [86].

### 3.6. Pathway Pathology and Treatment

The knowledge on molecular and cellular mechanisms provides a sound basis for the development of effective new therapies. Cancer immunotherapy may serve as a paradigm. The development of immune checkpoint inhibitors followed stringent research on the molecules providing stimulatory and inhibitory effects in the immune response [87,88]. For many effective therapies, the molecular mechanisms are known. However, for several others they are not; many facets still need to be resolved.

It is important to realize that with the identification of all protein-coding genes in the genome, we may not only find pathologies related to each of them, but all can now be approached as therapeutic targets. Any protein can be recognized and blocked by monoclonal antibodies. Genes with enzymatic activity may be inhibited by small molecules, and this is the reason why the 518 kinases of the genome (“kinome”) have gained much attention from researchers and pharmaceutical companies [89].

However, phosphorylation is only one of several posttranslational protein modifications that are important for regulating gene function. Others are, for instance, ubiquitinoylation (prior to proteasome degradation), ubiquitin-like protein conjugation by members of the ATG8 family for the initiation of autophagy-mediated degradation/recycling of organelles or bulk proteins, histone methylation, acetylation and deacetylation, etc. [90].

Histone modifications together with DNA methylation and demethylation constitute the blossoming field of epigenetics, which harbors great promise as these agents are able to modify gene expression genome-wide, thus acting similarly ubiquitously as transcription factors such as p53 or MYC. Not surprisingly, these types of genes are also frequently mutated in the cancer genome, constituting one major finding of the whole-genome mutation analysis studies within TCGA projects or by other efforts [91,92].

In the author’s view it is essential to test and find new combinations of drugs. Because many diseases are dependent on and driven by several pathways, they need to be approached by intelligent drug combinations. Cancer is a good example in this respect as it represents a malady that is usually driven by several genetic defects that are linked to different molecular and cellular pathways.

Combination therapy has become the standard for most successful treatments. The multibillion-dollar question is which combination should be tested. So far, there are only a few approaches with which to tackle this question. Pathway pathology may help in this regard as it first helps to identify the driving pathways of the disease process together with their mechanisms of action. Second, the pathway pathologist may then help the clinician to select a meaningful combination of drugs that interferes with the pathways in question.

For instance, the NFkB pathway is activated in many cancers, including the ones that are associated with an intratumoral inflammatory response. Hodgkin lymphoma is the prime example. The culprit NFkB pathway, however, is also related to drug resistance and tumor progression [90].

Glucocorticoids are effective in inhibiting this pathway and have been very successfully incorporated into the therapeutic regimens of Hodgkin lymphoma and non-Hodgkin lymphoma. In other cancer types, however, these drugs are not very popular, at least they do not seem to have been systematically tested.

Anti-inflammatory treatment, in general, appear to also be beneficial in other diseases, such as arteriosclerosis. Statins, for instance, are beneficial in secondary prevention of myocardial infarction primarily in individuals with elevated hsCRP levels. Although generally perceived as lipid-lowering medications, it is important to note that they are highly effective for reducing vascular events among apparently healthy individuals with low levels of LDL cholesterol but high serum CRP levels [93]. Interestingly, in the CANTOS trial using canakinumab, to target interleukin 1β, for the prevention of myocardial infarction in a high-risk population, not only were cardiovascular events significantly reduced, but the mortality of lung cancer also was diminished [94]. This was probably due to the beneficial effect of the anti-inflammatory action of canakinumab on incident, preexisting lung cancers in the study population.

Thus, it is very likely that anti-inflammatory agents are effective agents in cancer therapy, at least in those cancer types that are associated with inflammation. These cases can be identified by investigating clinical parameters of inflammation such as serum CRP, but also by the pathologist just looking at the tumor biopsy and evaluating the extent of inflammatory cell infiltrate. Needless to say, by characterizing the type of the inflammatory infiltrate, e.g., as lymphocytes, neutrophils, eosinophils, plasma cells, macrophages or mast cells, it is possible to decipher which cytokines and chemokines might be secreted by the tumor cells, and thus which molecular pathway of inflammation is activated by the malignancy.

Statins have also consistently been reported to be beneficial for cancer patients [95,96,97], and it is important to note that most of these anti-inflammatory agents have no or little side effects compared to conventional chemotherapeutic drugs.

### 3.7. Pathway Pathology of Anti-Cancer Drugs

Cell death and in particular apoptosis are the major cellular pathways activated by anti-cancer drugs. However, there are relatively few studies that systematically report on the molecular pathways activated by these drugs [98,99]. Cell death is a major cellular pathway in many human diseases and has been molecularly dissected in various subtypes, including apoptosis, necroptosis, immunogenic cell death, etc. [100]. Amazingly, it is only becoming slowly apparent which type of cell death is targeted by which antineoplastic drug and whether and how the cellular background and genetic makeup of the cancer cells influences the therapeutic outcome.

For instance, the antineoplastic action of oxaliplatin and anthracyclines is mediated mainly by immunogenic cell death [101]. This type of cell death is dependent on the functionality of the patient’s immune system. Many chemotherapeutic drugs, however, suppress the immune system via their antiproliferative activities. Thus, it is important to find the right balance in selecting different agents, and the important question therefore is “Are there any biomarkers that can predict the therapeutic response?”. The answer is yes, but any biomarker needs to be interpreted within its cellular and molecular context. Thus, what is needed is “pathway pathology”.

Bernard Weinstein coined the term “oncogene addiction” to describe the dependence of neoplasms on a single oncogene. The success of anti-EGFR therapy in lung adenocarcinoma was probably the stimulus for establishing this concept [102]. It seems however to be too restrictive; “pathway addition” is probably the better term. Another important concept for new therapies is “synthetic lethality”, stating that targeting the combination of deficiencies in the expression of two or more genes can lead to cell death. Even molecular targets such as MYC, that have been considered “undruggable” so far, may become treatable in the future [103]. Synthetic lethality is also the basis of the successful application of PARP inhibitors in BRCA-mutated ovarian carcinomas [104].

Pathway pathology is another concept that might be helpful in rationalizing and developing new combination therapies that are based on the intelligent interpretation of clinical, pathological and molecular findings of the disease within a specific patient.

### 3.8. Pathway Pathology—Is It Really New?

The answer is “Yes and No”. First of all, it is important to realize that the term “pathway” is used in multiple ways. So, it is important to delineate the similarities and differences in the usages of the term.

Pathway pathology should not be equated with pathway medicine in the sense of clinical pathways, i.e., algorithms that are applied in the clinical management of patients. Pathway medicine in this meaning is a simplistic and reductionist approach to solve medical problems. It suggests that medicine can be best handled by yes/no decisions. As already pointed out above, biology and biomedicine alike are not so easy and require a more differentiated analysis of the problem, taking into account gene dosage, biological functions and cellular networks.

Instead, pathway pathology is much more related to gene signaling pathways. It incorporates the fact that genes are members of a molecular network with several other constituents and often multiple biological functions. These functions have been addressed by a diverse terminology.

In cancer, the term “hallmarks” has been introduced and has become very popular [45,105]. It also represents, however, a somehow reductionist approach by stating that all cancer types can be described by a few hallmarks, i.e., sustained cell proliferation, evasion of growth suppression, activating invasion and metastasis, enabling replicative immortality, inducing angiogenesis and resisting cell death [105]. In the second edition of this landmark publication, Hanahan and Weinberg introduced additional emerging and enabling hallmarks: deregulating cellular energetics, avoiding immune destruction, tumor-promoting inflammation, genome instability and mutation. Importantly, the two publications propagated the fact that these hallmarks are associated with molecular signaling networks that can be specifically targeted for therapy [45].

The “Vogelgram” of colon cancer can be viewed as the first pathway pathology classification of a tumor disease, as it correlated specific genetic events with morphologically detectable steps in colorectal carcinogenesis, including biological features, in particular invasion and metastasis [106]. In later years Vogelstein reinforced the connection between genetic mutations and cellular processes being affected by them. He identified 12 cancer cell signaling pathways and cellular mechanisms (Notch, Hedgehog, APC, TGF-beta, MAPK, STAT, PI3K, RAS, chromatin modification, transcriptional regulation, cell cycle/apoptosis and DNA damage control) that he connected to three core cellular processes (cell survival, cell fate and genome maintenance) that all confer a selective growth advantage [58]. The cellular processes of Vogelstein correspond to the cellular pathways as outlined in this article. The Vogelstein approach may thus be viewed as a precursor to the concept of pathway pathology. There are still some important differences.

Firstly, both Hanahan and Weinberg, as well as Vogelstein et al., somehow oversimplified the complex biology and pathology of cancer. The pathological characterization of tumors includes many additional features that each represent specific cellular pathways that are mediated by specific molecular events, e.g., growth along nerves/perineural spread, lymphangiosis carcinomatosa (which is distinct from lymphonodular dissemination) or metaplastic differentiation, such as ossification. Some of these, namely perineural growth, have already been characterized molecularly [83], and it is clearly foreseeable that this will also happen to most other cellular pathways. The Nobel prizes for medicine and physiology, but also chemistry, are usually given to scientists that elucidate new cellular pathways and their molecular constituents, as exemplified by apoptosis (2002: Sidney Brenner, H. Robert Horvitz and John E. Sulston), autophagy (1974: Christian de Duve; 2016: Yoshinori Osuni) and DNA repair (2015: Tomas Lindahl, Paul Modrich and Aziz Sancar).

Secondly, Hanahan and Weinberg did not integrate pathology into their hallmark paper and Vogelstein did so only by using rather crude descriptors of pathology such as early and late adenoma or metastasis. Thirdly and most importantly, they applied their concepts “only” to tumor pathology. Although malignant tumors are highly complex biological systems and cancer has been entitled “The emperor of all maladies” [107], they do not represent the only diseases of mankind. In fact, all are governed by cells of origin as well as cellular and molecular pathways. Therefore, pathway pathology is a much broader approach that is applicable for any malady, and in fact it is highly interesting and instructive to see that similar pathomechanisms and signaling pathways apply to many different diseases.

For instance, NFkB is essential in cancer but also inflammation. Apoptosis and cell death is essential for many non-cancer maladies, including Alzheimer’s, Parkinson’s and myocardial infarction. Pathway pathology may be viewed as the resurgence of general pathology, entrenching the knowledge of whole-genome genetics and molecular cellular biology.

## 4. Outlook

Biomedical research and clinical practice are experiencing fundamental changes and important challenges. Digitalization and artificial intelligence will become constant companions as much more data and information need to be analyzed and incorporated into the routine work of researchers and medical doctors. However, it is the author’s belief that an “educated brain” will remain fundamental for classifying diseases and improving the treatment of patients. The future will tell whether or not the pathway pathology concept will be helpful in this regard.

## Figures and Tables

**Table 1:** Comparison of ICD-11 and ICD-10 chapters and codes.

ICD-11			ICD-10		
No.	Title	Codes	No.	Title	Codes
1	Certain infectious and parasitic diseases	1A00-1G8Y	1	Certain infectious and parasitic diseases	A00-B99
2	Neoplasms	2A00-2F9Y	2	Neoplasms	C00-D48
3	Diseases of blood and blood-forming organs	3A00-3C0Z	3	Diseases of the blood and blood-forming organs in addition to certain disorders involving the immune system	D50-D89
4	Disorders of the immune system	4A00-4B4Z	4	Endocrine, nutritional and metabolic diseases	E00-E90
5	Endocrine, nutritional or metabolic diseases	5A00-5B3Z	5	Mental and behavioral disorders	F00-F99
6	Mental, behavioral or neurodevelopmental disorders	6A00-6E8Z	6	Diseases of the nervous system	G00-G99
7	Sleep–wake disorders	7A00-7B2Z	7	Diseases of the eye and adnexa	H00-H59
8	Diseases of the nervous system	8A00-8E7Z	8	Diseases of the ear and mastoid process	H60-H95
9	Diseases of the visual system	9A00-9A4Z	9	Diseases of the circulatory system	I00-I99
10	Diseases of the ear and mastoid process	AA00-AC0Z	10	Diseases of the respiratory system	J00-J99
11	Diseases of the circulatory system	BA00-BE2Z	11	Diseases of the digestive system	K00-K93
12	Diseases of the respiratory system	CA00-CB7Z	12	Diseases of the skin and subcutaneous tissue	L00-L99
13	Diseases of the digestive system	DA00-DE2Z	13	Diseases of the musculoskeletal system and connective tissue	M00-M99
14	Diseases of the skin	EA00-EM0Z	14	Diseases of the genitourinary system	N00-N99
15	Diseases of the musculoskeletal system or connective tissue	FA00-FC0Z	15	Pregnancy, childbirth and the puerperium	O00-O99
16	Diseases of the genitourinary system	GA00-GC8Z	16	Certain conditions originating in the perinatal period	P00-P96
17	Conditions related to sexual health	HA00-HA8Z	17	Congenital malformations, deformations and chromosomal abnormalities	Q00-Q99
18	Pregnancy, childbirth and the puerperium	JA00-JB6Z	18	Symptoms, signs and abnormal clinical and laboratory findings, not classified elsewhere	R00-R99
19	Certain conditions originating in the perinatal period	KA00-KA0Z	19	Injury, poisoning and certain other consequences of external causes	S00-T98
20	Developmental anomalies	LA00-LD0Z	20	External causes of morbidity and mortality	V01-Y98
21	Symptoms, signs or clinical findings, not classified elsewhere	MA00-MH2Y	21	Factors influencing health status and contact with health services	Z00-Z99
22	Injury, poisoning or certain other consequences of external causes	NA00-NF2Z	22	Codes for special purposes	U00-U99
23	External causes of morbidity or mortality	PA00-PL2Z			
24	Factors influencing health status or contact with health services	QA00-QF4Z			
25	Codes for special purposes	RA00-RA26			
26	Supplementary chapter: traditional medicine conditions	TM1-SJ3Z			
27	V—supplementary section for functioning assessment	VA00-VB40.Z			
28	X—extension codes	XS8H-XD19J4			

**Table 2:** List of fields of the ICD-11 content model.

1	ICD entry title
2	Classification properties
3	Textual definition
4	Terms
5	Body system/structure description
6	Temporal properties
7	Severity of subtype properties
8	Manifestation properties
9	Causal properties
10	Functioning properties
11	Specific condition properties
12	Treatment properties
13	Diagnostic criteria
Parameters 1–7 are essential requirements.	

## References

[1] Mattson M.P. (2014). Superior pattern processing is the essence of the evolved human brain. Front. Neurosci..

[2] Aisenberg A.C. (2000). Historical review of lymphomas. Br. J. Haematol..

[3] Jaffe E.S., Harris N.L., Stein H., Isaacson P.G. (2008). Classification of lymphoid neoplasms: The microscope as a tool for disease discovery. Blood.

[4] Etzel B.-M., Gerth M., Chen Y., Wünsche E., Facklam T., Beck J.F., Guntinas-Lichius O., Petersen I. (2017). Mutation analysis of tumor necrosis factor alpha-induced protein 3 gene in Hodgkin lymphoma. Pathol.-Res. Pr..

[5] Nagel D., Vincendeau M., Eitelhuber A.C., Krappmann D. (2014). Mechanisms and consequences of constitutive NF-κB activation in B-cell lymphoid malignancies. Oncogene.

[6] Petersen I., Kotb W.F.A., Friedrich K.-H., Schlüns K., Böcking A., Dietel M. (2009). Core classification of lung cancer: Correlating nuclear size and mitoses with ploidy and clinicopathological parameters. Lung Cancer.

[7] Bockmühl U., Schwendel A., Dietel M., Petersen I. (1996). Distinct patterns of chromosomal alterations in high- and low-grade head and neck squamous cell carcinomas. Cancer Res..

[8] Petersen I., Petersen S. (2001). Towards a Genetic-Based Classification of Human Lung Cancer. Anal. Cell. Pathol..

[9] Ried T., Petersen I., Holtgreve-Grez H., Speicher M., Schröck E., Du Manoir S., Cremer T. (1994). Mapping of multiple DNA gains and losses in primary small cell lung carcinomas by comparative genomic hybridization. Cancer Res..

[10] Garber M.E., Troyanskaya O.G., Schluens K., Petersen S., Thaesler Z., Pacyna-Gengelbach M., van de Rijn M., Rosen G.D., Perou C., Whyte R.I. (2001). Diversity of gene expression in adenocarcinoma of the lung. Proc. Natl. Acad. Sci. USA.

[11] Perou C., Jeffrey S., van de Rijn M., Rees C.A., Eisen M., Ross D.T., Pergamenschikov A., Williams C.F., Zhu S.X., Lee J.C.F. (1999). Distinctive gene expression patterns in human mammary epithelial cells and breast cancers. Proc. Natl. Acad. Sci. USA.

[12] Capper D., Jones D.T.W., Sill M., Hovestadt V., Schrimpf D., Sturm D., Koelsche C., Sahm F., Chavez L., Reuss D.E. (2018). DNA methylation-based classification of central nervous system tumours. Nature.

[13] Koelsche C., Schrimpf D., Stichel D., Sill M., Sahm F., Reuss D.E., Blattner M., Worst B., Heilig C.E., Beck K. (2021). Sarcoma classification by DNA methylation profiling. Nat. Commun..

[14] Petersen I. (2017). Entitäten der Weichteilsarkome. Trauma Berufskrankh..

[15] Dietel M., Jöhrens K., Laffert M.V., Hummel M., Bläker H., Pfitzner B.M., Lehmann A., Denkert C., Darb-Esfahani S., Lenze D. (2015). A 2015 update on predictive molecular pathology and its role in targeted cancer therapy: A review focussing on clinical relevance. Cancer Gene Ther..

[16] Tsimberidou A.M., Fountzilas E., Nikanjam M., Kurzrock R. (2020). Review of precision cancer medicine: Evolution of the treatment paradigm. Cancer Treat. Rev..

[17] Clamp M., Fry B., Kamal M., Xie X., Cuff J., Lin M.F., Kellis M., Lindblad-Toh K., Lander E.S. (2007). Distinguishing protein-coding and noncoding genes in the human genome. Proc. Natl. Acad. Sci. USA.

[18] Kottke J. (2010). How Many Words do Shakespeare Know. https://kottke.org/10/04/how-many-words-did-shakespeare-know.

[19] du Plessis L., Škunca N., Dessimoz C. (2011). The what, where, how and why of gene ontology—A primer for bioinformaticians. Briefings Bioinform..

[20] Lancellotta V., Guinot J.L., Fionda B., Rembielak A., Di Stefani A., Gentileschi S., Federico F., Rossi E., Guix B., Chyrek A.J. (2020). SKIN-COBRA (Consortium for Brachytherapy data Analysis) ontology: The first step towards interdisciplinary standardized data collection for personalized oncology in skin cancer. J. Contemp. Brachyther..

[21] Tagliaferri L., Kovács G., Autorino R., Budrukkar A., Guinot J.L., Hildebrand G., Johansson B., Monge R.M., Meyer J.E., Niehoff P. (2016). ENT COBRA (Consortium for Brachytherapy Data Analysis): Interdisciplinary standardized data collection system for head and neck patients treated with interventional radiotherapy (brachytherapy). J. Contemp. Brachyther..

[22] Aymé S., Bellet B., Rath A. (2015). Rare diseases in ICD11: Making rare diseases visible in health information systems through appropriate coding. Orphanet J. Rare Dis..

[23] Ribatti D. (2019). Rudolf Virchow, the founder of cellular pathology. Rom. J. Morphol. Embryol..

[24] Dawood S., Austin L., Cristofanilli M. (2014). Cancer stem cells: Implications for cancer therapy. Oncology.

[25] Yamanaka S. (2009). A Fresh Look at iPS Cells. Cell.

[26] Hayflick L., Stinebring W.R. (1955). Intracellular growth of Pleuropneumonia-like organisms. Anatomincal. Record..

[27] Hayflick L., Stinebring W.R. (1960). Intracellular Growth of Pleuropneumonialike Organisms (PPLO) in Tissue Culture and in OVO*. Ann. N. Y. Acad. Sci..

[28] Hayflick L., Chanock R.M. (1965). Mycoplasma Species of Man. Bacteriol. Rev..

[29] Hayflick L., Moorhead P.S. (1961). The serial cultivation of human diploid cell strains. Exp. Cell Res..

[30] Ben-David U., Amon A. (2020). Context is everything: Aneuploidy in cancer. Nat. Rev. Genet..

[31] Poulin E.J., Bera A.K., Lu J., Lin Y.-J., Strasser S.D., Paulo J.A., Huang T.Q., Morales C., Yan W., Cook J. (2019). Tissue-Specific Oncogenic Activity of KRASA146T. Cancer Discov..

[32] Hirata E., Sahai E. (2017). Tumor Microenvironment and Differential Responses to Therapy. Cold Spring Harb. Perspect. Med..

[33] Hayflick L., Norton T.W., Plotkin S.A., Koprowski H. (1962). Preparation of Polio vaccines in a human fetal diploid cell strain. Am. J. Hygiene.

[34] Olshansky S.J., Hayflick L. (2017). The Role of the WI-38 Cell Strain in Saving Lives and Reducing Morbidity. AIMS Public Health.

[35] Rodriguez-Galindo C., Allen C.E. (2020). Langerhans cell histiocytosis. Blood.

[36] Ashburner M., Ball C.A., Blake J.A., Botstein D., Butler H., Cherry J.M., Davis A.P., Dolinski K., Dwight S.S., Eppig J.T. (2000). Gene Ontology: Tool for the unification of biology. Nat. Genet..

[37] Kanehisa M., Furumichi M., Tanabe M., Sato Y., Morishima K. (2017). KEGG: New perspectives on genomes, pathways, diseases and drugs. Nucleic Acids Res..

[38] Hayflick L. (1965). The limited in vitro lifetime of human diploid cell strains. Exp. Cell Res..

[39] Zhao S., Wang F., Liu L. (2019). Alternative Lengthening of Telomeres (ALT) in Tumors and Pluripotent Stem Cells. Genes.

[40] Muñoz-Espín D., Cañamero M., Maraver A., López G.G., Contreras J., Murillo-Cuesta S., Rodríguez-Baeza A., Varela-Nieto I., Ruberte J., Collado M. (2013). Programmed Cell Senescence during Mammalian Embryonic Development. Cell.

[41] Muñoz-Espín D., Serrano M. (2014). Cellular senescence: From physiology to pathology. Nat. Rev. Mol. Cell Biol..

[42] Storer M., Mas A., Robert-Moreno À., Pecoraro M., Ortells M.C., Di Giacomo V., Yosef R., Pilpel N., Krizhanovsky V., Sharpe J. (2013). Senescence Is a Developmental Mechanism that Contributes to Embryonic Growth and Patterning. Cell.

[43] Serrano M., Lin A.W., McCurrach M.E., Beach D., Lowe S.W. (1997). Oncogenic ras Provokes Premature Cell Senescence Associated with Accumulation of p53 and p16INK4a. Cell.

[44] Collado M., Serrano M. (2010). Senescence in tumours: Evidence from mice and humans. Nat. Rev. Cancer.

[45] Hanahan D., Weinberg R.A. (2011). Hallmarks of Cancer: The Next Generation. Cell.

[46] Nielsen T.O., Poulin N.M., Ladanyi M. (2015). Synovial Sarcoma: Recent Discoveries as a Roadmap to New Avenues for Therapy. Cancer Discov..

[47] Sauer C.M., Haugg A.M., Chteinberg E., Rennspiess D., Winnepenninckx V., Speel E.-J., Becker J.C., Kurz A.K., Hausen A.Z. (2017). Reviewing the current evidence supporting early B-cells as the cellular origin of Merkel cell carcinoma. Crit. Rev. Oncol..

[48] Dammert M.A., Brägelmann J., Olsen R.R., Böhm S., Monhasery N., Whitney C.P., Chalishazar M.D., Tumbrink H.L., Guthrie M.R., Klein S. (2019). MYC paralog-dependent apoptotic priming orchestrates a spectrum of vulnerabilities in small cell lung cancer. Nat. Commun..

[49] Xia Y., Zhang X. (2020). The Spectrum of MYC Alterations in Diffuse Large B-Cell Lymphoma. Acta Haematol..

[50] Xu W., Yang Z., Xie C., Zhu Y., Shu X., Zhang Z., Li N., Chai N., Zhang S., Wu K. (2018). PTEN lipid phosphatase inactivation links the hippo and PI3K/Akt pathways to induce gastric tumorigenesis. J. Exp. Clin. Cancer Res..

[51] Travis W.D., Brambilla E., Noguchi M., Nicholson A.G., Geisinger K.R., Yatabe Y., Beer D.G., Powell C., Riely G.J., Van Schil P.E. (2011). International Association for the Study of Lung Cancer/American Thoracic Society/European Respiratory Society International Multidisciplinary Classification of Lung Adenocarcinoma. J. Thorac. Oncol..

[52] Sinn P., Aulmann S., Wirtz R., Schott S., Marmé F., Varga Z., Lebeau A., Kreipe H., Schneeweiss A. (2013). Multigene Assays for Classification, Prognosis, and Prediction in Breast Cancer: A Critical Review on the Background and Clinical Utility. Geburtshilfe Frauenheilkd..

[53] Moran S., Cardus A.M., Sayols S., Musulen E., Balaña C., Estival-Gonzalez A., Moutinho C., Heyn H., Diaz-Lagares A., de Moura M.C. (2016). Epigenetic profiling to classify cancer of unknown primary: A multicentre, retrospective analysis. Lancet Oncol..

[54] Duruisseaux M., Martínez-Cardús A., Calleja-Cervantes M.E., Moran S., de Moura M.C., Davalos V., Piñeyro D., Sanchez-Cespedes M., Girard N., Brevet M. (2018). Epigenetic prediction of response to anti-PD-1 treatment in non-small-cell lung cancer: A multicentre, retrospective analysis. Lancet Respir. Med..

[55] Petersen I. (2018). Predictive pathology of lung cancer immunotherapy response. Lancet Respir. Med..

[56] Kamps R., Brandão R.D., Bosch B.J.V.D., Paulussen A.D.C., Xanthoulea S., Blok M.J., Romano A. (2017). Next-Generation Sequencing in Oncology: Genetic Diagnosis, Risk Prediction and Cancer Classification. Int. J. Mol. Sci..

[57] Chan T., Yarchoan M., Jaffee E., Swanton C., Quezada S., Stenzinger A., Peters S. (2019). Development of tumor mutation burden as an immunotherapy biomarker: Utility for the oncology clinic. Ann. Oncol..

[58] Vogelstein B., Papadopoulos N., Velculescu V.E., Zhou S., Diaz L.A., Kinzler K.W. (2013). Cancer Genome Landscapes. Science.

[59] Zhang Z., Li H., Jiang S., Li R., Li W., Chen H., Bo X. (2019). A survey and evaluation of Web-based tools/databases for variant analysis of TCGA data. Briefings Bioinform..

[60] Hainaut P., Pfeifer G.P. (2016). SomaticTP53Mutations in the Era of Genome Sequencing. Cold Spring Harb. Perspect. Med..

[61] Petersen I., Ohgaki H., Ludeke B.I., Kleihues P. (1993). p53 mutations in phenacetin-associated human urothelial carcinomas. Carcinogenesis.

[62] Koshland D. (1993). Molecule of the year. Science.

[63] Soussi T. (2010). The history of p53. EMBO Rep..

[64] Dolgin E. (2017). The most popular genes in the human genome. Nat. Cell Biol..

[65] Stockwell B.R., Angeli J.P.F., Bayir H., Bush A.I., Conrad M., Dixon S.J., Fulda S., Gascón S., Hatzios S.K., Kagan V.E. (2017). Ferroptosis: A Regulated Cell Death Nexus Linking Metabolism, Redox Biology, and Disease. Cell.

[66] Bykov V.N., Wiman K.G. (2014). Mutant p53 reactivation by small molecules makes its way to the clinic. FEBS Lett..

[67] Gurpinar E., Vousden K.H. (2015). Hitting cancers’ weak spots: Vulnerabilities imposed by p53 mutation. Trends Cell Biol..

[68] Muller P.A., Vousden K.H. (2014). Mutant p53 in Cancer: New Functions and Therapeutic Opportunities. Cancer Cell.

[69] Parrales A., Iwakuma T. (2015). Targeting Oncogenic Mutant p53 for Cancer Therapy. Front. Oncol..

[70] Brosh R., Rotter V. (2009). When mutants gain new powers: News from the mutant p53 field. Nat. Rev. Cancer.

[71] Green D.R., Kroemer G. (2009). Cytoplasmic functions of the tumour suppressor p53. Nat. Cell Biol..

[72] Mertens F., Antonescu C.R., Mitelman F. (2016). Gene fusions in soft tissue tumors: Recurrent and overlapping pathogenetic themes. Genes Chromosom. Cancer.

[73] Canale M., Petracci E., Delmonte A., Bronte G., Chiadini E., Ludovini V., Dubini A., Papi M., Baglivo S., De Luigi N. (2020). Concomitant TP53 Mutation Confers Worse Prognosis in EGFR-Mutated Non-Small Cell Lung Cancer Patients Treated with TKIs. J. Clin. Med..

[74] Schulze S., Petersen I. (2011). Gender and ploidy in cancer survival. Cell. Oncol..

[75] Von Mehren M., Joensuu H. (2018). Gastrointestinal Stromal Tumors. J. Clin. Oncol..

[76] Pietas A., Schlüns K., Marenholz I., Schafer B., Heizmann C.W., Petersen I. (2002). Molecular Cloning and Characterization of the Human S100A14 Gene Encoding a Novel Member of the S100 Family. Genomics.

[77] Brettmann E., Strong C.D.G. (2018). Recent evolution of the human skin barrier. Exp. Dermatol..

[78] Klebig C., Seitz S., Arnold W., Deutschmann N., Pacyna-Gengelbach M., Scherneck S., Petersen I. (2005). Characterization of {gamma}-aminobutyric acid type A receptor-associated protein, a novel tumor suppressor, showing reduced expression in breast cancer. Cancer Res..

[79] Salah F., Ebbinghaus M., Muley V.Y., Zhou Z., Al-Saadi K.R.D., Pacyna-Gengelbach M., O’Sullivan G.A., Betz H., König R., Wang Z.-Q. (2016). Tumor suppression in mice lacking GABARAP, an Atg8/LC3 family member implicated in autophagy, is associated with alterations in cytokine secretion and cell death. Cell Death Dis..

[80] Chavez-Dominguez R., Perez-Medina M., Lopez-Gonzalez J.S., Galicia-Velasco M., Aguilar-Cazares D. (2020). The Double-Edge Sword of Autophagy in Cancer: From Tumor Suppression to Protumor Activity. Front. Oncol..

[81] Petersen I., Dietel M., Geilenkeuser W.J., Mireskandari M., Weichert W., Steiger K., Scheel A.H., Büttner R., Schirmacher P., Warth A. (2017). EGFR immunohistochemistry as biomarker for antibody-based therapy of squamous NSCLC—Experience from the first ring trial of the German Quality Assurance Initiative for Pathology (QuIP®). Pathol.-Res. Pr..

[82] Zayed H., Petersen I. (2018). Stem cell transcription factor SOX2 in synovial sarcoma and other soft tissue tumors. Pathol.-Res. Pr..

[83] Amit M., Na’Ara S., Gil Z. (2016). Mechanisms of cancer dissemination along nerves. Nat. Rev. Cancer.

[84] Chiang S.P.H., Cabrera R.M., Segall J.E. (2016). Tumor cell intravasation. Am. J. Physiol. Cell Physiol..

[85] Kotb W.F.A., Petersen I. (2012). Morphology, DNA ploidy and HPV in lung cancer and head and neck cancer. Pathol.-Res. Pr..

[86] Cosenza M.R., Krämer A. (2015). Centrosome amplification, chromosomal instability and cancer: Mechanistic, clinical and therapeutic issues. Chromosom. Res..

[87] Iwai Y., Hamanishi J., Chamoto K., Honjo T. (2017). Cancer immunotherapies targeting the PD-1 signaling pathway. J. Biomed. Sci..

[88] Wei S.C., Duffy C.R., Allison J.P. (2018). Fundamental Mechanisms of Immune Checkpoint Blockade Therapy. Cancer Discov..

[89] Berard A., Kroeker A., McQueen P., Coombs K.M. (2018). Methods and approaches to disease mechanisms using systems kinomics. Synth. Syst. Biotechnol..

[90] Narayanan S., Cai C.-Y., Assaraf Y.G., Guo H.-Q., Cui Q., Wei L., Huang J.-J., Ashby C.R., Chen Z.-S. (2020). Targeting the ubiquitin-proteasome pathway to overcome anti-cancer drug resistance. Drug Resist. Updat..

[91] Fernandez-Cuesta L., Peifer M., Lu X., Sun R., Ozretić L., Seidel D., Zander T., Leenders F., George J., Müller C. (2014). Frequent mutations in chromatin-remodelling genes in pulmonary carcinoids. Nat. Commun..

[92] Lee M.P. (2019). Understanding Cancer Through the Lens of Epigenetic Inheritance, Allele-Specific Gene Expression, and High-Throughput Technology. Front. Oncol..

[93] Aday A., Ridker P.M. (2018). Antiinflammatory Therapy in Clinical Care: The CANTOS Trial and Beyond. Front. Cardiovasc. Med..

[94] Ridker P.M., MacFadyen J.G., Thuren T., Everett B.M., Libby P., Glynn R.J., Lorenzatti A., Krum H., Varigos J., Siostrzonek P. (2017). Effect of interleukin-1β inhibition with canakinumab on incident lung cancer in patients with atherosclerosis: Exploratory results from a randomised, double-blind, placebo-controlled trial. Lancet.

[95] Hindler K., Cleeland C.S., Rivera E., Collard C.D. (2006). The Role of Statins in Cancer Therapy. Oncologist.

[96] Ling Y., Yang L., Huang H., Hu X., Zhao C., Huang H., Ying Y. (2015). Prognostic Significance of Statin Use in Colorectal Cancer. Medicine.

[97] Moon D.C., Lee H.S., Lee Y.I., Chung M.J., Park J.Y., Park S.W., Song S.Y., Chung J.B., Bang S. (2016). Concomitant Statin Use Has a Favorable Effect on Gemcitabine-Erlotinib Combination Chemotherapy for Advanced Pancreatic Cancer. Yonsei Med. J..

[98] Dasari S., Tchounwou P.B. (2014). Cisplatin in cancer therapy: Molecular mechanisms of action. Eur. J. Pharmacol..

[99] Tchounwou P.B., Dasari S., Noubissi F.K., Ray P., Kumar S. (2021). Advances in Our Understanding of the Molecular Mechanisms of Action of Cisplatin in Cancer Therapy. J. Exp. Pharmacol..

[100] Galluzzi L., Vitale I., Aaronson S.A., Abrams J.M., Adam D., Agostinis P., Alnemri E.S., Altucci L., Amelio I., Andrews D.W. (2018). Molecular mechanisms of cell death: Recommendations of the Nomenclature Committee on Cell Death 2018. Cell Death Differ..

[101] Kroemer G., Galluzzi L., Kepp O., Zitvogel L. (2013). Immunogenic Cell Death in Cancer Therapy. Annu. Rev. Immunol..

[102] Weinstein I.B., Joe A. (2008). Oncogene Addiction. Cancer Res..

[103] Wang C., Fang H., Zhang J., Gu Y. (2021). Targeting “undruggable” c-Myc protein by synthetic lethality. Front. Med..

[104] Pilié P.G., Gay C.M., Byers L.A., O’Connor M.J., Yap T.A. (2019). PARP Inhibitors: Extending Benefit Beyond BRCA-Mutant Cancers. Clin. Cancer Res..

[105] Hanahan D., Weinberg R.A. (2000). The Hallmarks of Cancer. Cell.

[106] Fearon E.R., Vogelstein B. (1990). A genetic model for colorectal tumorigenesis. Cell.

[107] Mukherjee S. (2011). The Emperor of All Maladies.

